# It’s more than just lubrication of the skin: parents’ experiences of caring for a child with ichthyosis

**DOI:** 10.1080/21642850.2022.2053685

**Published:** 2022-04-05

**Authors:** Elisabeth Daae, Kristin Billaud Feragen, Jan C. Sitek, Charlotte von der Lippe

**Affiliations:** a Center for Rare Disorders, Oslo University Hospital HF, Oslo, Norway; b Department of Dermatology, Oslo University Hospital HF, Oslo, Norway

**Keywords:** Ichthyosis, skin disorder, parents, qualitative, rare disease

## Abstract

**Background**: The ichthyoses are a group of genetic skin disorders, characterized by excessive amounts of dry, thickened skin, which may be fragile, inelastic and prone to fissures and infection. Skin care is time consuming and demanding, and, usually performed by the parents.

**Methods**: We aimed to explore parental experience of caring for a child with ichthyosis, and collected data using semistructured interview, and thematic analysis.

**Results**: Our analysis revealed four main themes: Parents' and others' reactions to the child's difference, Experiences with healthcare services, It's all skin care, and Impact on relationships.

**Conclusion**: After birth of a child with severe ichthyosis, the parents experienced emotional distress and stigmatization due to the different appearance of the skin and healthcare professionals' lack of knowledge. Skin care caused pain in the child, was time consuming, and caused financial burdens. This study can guide healthcare professionals on where to focus future efforts in meeting the clinical and psychological needs of parents caring for a child with ichthyosis.

## Introduction

The skin, which is the largest of our sense organs, is the first organ to develop in fetal life and the main organ for tactile interaction (Chuong et al., 2002). Skin-to-skin contact immediately after birth is important for the child and the mother (Moore, Bergman, Anderson, & Medley, 2016), and its positive effects have been well documented in the literature (WHO immediate KMC Group, 2021). However, when a child is born with a skin condition such as ichthyosis, basic skin-to-skin contact, everyday life, and parental care may be a challenge from birth and early on.

The inherited congenital ichthyoses constitute a heterogenic group of genetic skin disorders, divided into non-syndromic and syndromic congenital ichthyosis (Traupe, Fischer, & Oji, 2014; Yoneda, 2016). Ichthyosis is characterized by persistently dry, thickened, and scaly skin (Traupe et al., 2014), and typically presents itself at birth or within the initial years of life. It is difficult to differentiate between the ichthyosis subtypes during the neonatal period (Dyer, Spraker, & Williams, 2013). The mode of inheritance may be autosomal dominant, autosomal recessive, or X-linked (Mazereeuw-Hautier et al., 2018). Except for the mild dominant form, ichthyosis vulgaris, which has a prevalence of 1:250–1000 (Oji et al., 2010), most congenital ichthyoses are rare. X-linked ichthyosis has a prevalence of 1:6000 (Richard, 2017). Autosomal recessive congenital ichthyoses (ARCIs), which include lamellar ichthyosis, congenital ichthyosiform erythroderma, and harlequin ichthyosis, are even rarer, with overall incidence estimated to be approximately 1 in 200,000 births (Richard, 2017). In Scandinavia, ARCI are estimated to have a prevalence of 1:100,000 (Pigg et al., 2016).

Almost the entire skin is affected in nearly all types of ichthyosis. The appearance of the skin may vary, from presenting fine and white scales to being dark and brown (Oji et al., 2010). The skin can become extremely fragile in some types of ichthyosis and can be damaged by even minor friction or trauma (Takeichi & Akiyama, 2016). In the more severe forms of ichthyosis, the loss of moisture in the skin may lead to it becoming extremely dry, inelastic, and tight, which makes movements uncomfortable (Mazereeuw-Hautier et al., 2018). In addition, fissure formation and erosion of the skin may lead to harmful skin infections (Vahlquist, Fischer, & Torma, 2018). Currently, there is no cure for ichthyosis, but treatment may alleviate the symptoms (Mazereeuw-Hautier et al., 2018). Treatment includes daily emollient baths in salty/oily water, exfoliating excessive scales, and applying cream/lotion/ointment several times a day to rehydrate the skin. Skin care is time consuming and often performed by the child’s parents (Dreyfus et al., 2015).

Pediatric chronic illnesses affect the child and the entire family (Lim & Zebrack, 2004). The management of chronic conditions have gradually moved from hospital-based care to home-based care, which entails that the parents are key members of their children’s healthcare teams as well as their primary caregivers (Kepreotes, Keatinge, & Stone, 2010). As a consequence, parents may experience a higher level of caregiver challenges than parents of healthy children (Cousino & Hazen, 2013; Stabile & Allin, 2012). Adapting to their child’s diagnosis and the associated daily caregiver challenges becomes essential for minimizing emotional distress and facilitating coping efforts (Cousino & Hazen, 2013).

The Dermatology Life Quality Index (DLQI) scores place ichthyoses among skin disorders that have the most harmful impact on a patient’s quality of life (Bodemer et al., 2011). Despite this, only a few studies have investigated health-related quality of life (HRQoL) for people with ichthyosis (Troiano & Lazzeri, 2020); even fewer studies have explored how parents adapt to the many challenges associated with their child’s diagnosis. A small number of studies have addressed the difficulties faced by the parents of children suffering from another skin disorder, epidermolysis bullosa, which also involves painful and time-consuming skin care (Kearney, Donohoe, & McAuliffe, 2020; Van Scheppingen, Lettinga, Duipmans, Maathuis, & Jonkman, 2008). A Swedish study showed that HRQoL was impaired for both children with congenital ichthyosis and their parents (Gånemo, 2010). The older children in this study reported their childhood being the most problematic period of their lives due to skin problems such as thick scaling, fissures, wounds, and pain. Individuals with severe skin disorders, such as ichthyosis or epidermolysis bullosa, may also present certain psychological symptoms, such as depression and/or anxiety, or experience harassment, which may be associated with their different appearance and uncomfortable skin symptoms (Dures, Morris, Gleeson, & Rumsey, 2011; Sun, Ren, Zaki, Maciejewski, & Choate, 2020).

As children with ichthyosis depend on their parents for care, understanding how parents perceive illness-related demands is important to shed light on the factors that affect parental stresses and strains. A substantial amount of evidence indicates that helping parents manage stress may lead to better health outcomes for children with chronic disorders (Senger, Ward, Barbosa-Leiker, & Bindler, 2016). Hence, understanding parents’ experiences may help achieve better health outcomes for children with chronic disorders, such as ichthyosis, and potentially improve their quality of life (Moola, 2012).

Qualitative research, such as the current study, provides insight into the psychological and psychosocial implications of parenting a child with ichthyosis. The aim of the study is to gain a deeper understanding of the subjective experiences of parents of children with ichthyosis, with the intention to identify challenges related to their child’s care and potential unmet needs.

## Methods

### Ethical considerations

The conduct, design, and reporting of this study follows the Standards for Reporting Qualitative Research (O’Brien, Harris, Beckman, Reed, & Cook, 2014). The study was conducted in accordance with the Helsinki Declaration. Ethical approval for the study was obtained from the Ethics Committee (Country, reference number 2019/567), and it was accepted by the Oslo University Hospital’s Data Protection Office (reference number 19/08354). All parents were required to provide written consent to participate. In addition, individuals with ichthyosis who were 16 years and older provided written consent for us to invite their parents to participate. To protect privacy and maintain anonymity, information regarding gender, exact age, and the number of children with the same diagnosis in a family is not provided. For the same reasons, we refer to the diagnoses as severe or mild ichthyosis without elaborating on the precise ichthyosis diagnosis.

### Methodological foundation

The use of semi-structured interviews allowed a systematic exploration of topics using predetermined open-ended questions and participant-led exploration (DiCicco-Bloom & Crabtree, 2006). Qualitative research generally involves exploring, understanding, and describing the personal and social experiences of participants and trying to capture their understanding of the relevant phenomena (Gray, 2018).

### Recruitment

The Center for Rare Disorders (CRD), Oslo University Hospital, Norway is a multidisciplinary resource center with a national responsibility for more than 100 disorders, including ichthyosis. The CRD has a voluntary patient registry that includes 29 individuals with congenital ichthyosis under the age of 16 and 70 individuals 16 years or older. The parents (mothers and fathers) of children under the age of 16 and with ichthyosis (*n *= 29) received an invitation by mail to participate in the study in August, 2019. A reminder letter was sent to 19 of the non-responding parents in October, 2019. In addition, 44 letters were sent to individuals with ichthyosis born between 1980 and 2003, asking for their consent to invite their parents to participate in the study. Adults born prior to 1980 were not invited, since older participant’s experiences were expected to be less relevant for informing today’s healthcare practices. Social media, the Department of Dermatology, OsloUniversity Hospital, and the Norwegian ichthyosis patient organization helped spread information about the study.

### Sample characteristics

A total of 19 parents (12 mothers and seven fathers) of 19 children agreed to participate in the study. For four families, both parents agreed to attend. Some parents had more than one child with ichthyosis. One couple chose to be interviewed together, while others were interviewed individually. Two of the children had syndromic ichthyosis, and 17 had non-syndromic forms, twelve had the severe form, and seven had the mild variant. Fourteen of the children were under the age of 15, and five were between 16 and 30 years old. See Table 1 for an overview of the participants.

**Table 1.** The participants.

Participant number	Mother (M)/father (F)	Age group of the child	Mild/Severe ichthyosis
1	Mother	0–15	Severe
2	Mother	16–30	Mild
3	Mother	16–30	Severe
4	Father	0–15	Severe
5	Mother	0–15	Mild
6	Mother	0–15	Mild
7	Mother	0–15	Mild
8	Mother	0–15	Severe
9	Father	0–15	Mild
10	Father	0–15	Severe
11	Father	0–15	Severe
12	Father	0–15	Severe
13	Mother	0–15	Severe
14	Mother	16–30	Severe
15/16	Mother and father	0–15	Severe
17	Mother	16–30	Severe
18	Mother	16–30	Mild
19	Father	0–15	Mild

### Data collection

The semi-structured interview guide was informed by previous literature and developed by the authors based on inputs from the patient representatives. The interview guide included open questions, such as asking them about their experiences of the following events: the pregnancy and birth, the initial time after birth, when the child was diagnosed, touching the baby’s skin, cuddling with the baby, and skin care. A pilot interview was conducted with a member of the ichthyosis patient organization (parent) to collect feedback regarding the questions and how the interview was perceived. The interview guide was slightly modified according to this feedback, and an extra question about the parents’ experience with touching their child’s skin was added. A flexible interview guide was used to ensure consistency across all interviews while simultaneously allowing participants to express themselves freely. Follow-up questions were asked to clarify answers or seek elaboration upon responses. The interviews were conducted by the first author from January to August 2020. Due to the Covid-19 situation, the interviews were conducted over the phone. On average, the interviews lasted for 82 minutes (range: 57–126 minutes) and were audio-recorded and transcribed verbatim.

### Data analysis

We employed a reflexive thematic analysis using the six phases outlined by Braun and Clarke (2006, 2019). The interviews were coded on a semantic level (inductive data-driven codes using the participants’ own words). Both the first and the last author coded the interviews individually to ensure credibility and dependability. The codes were classified into main themes and subthemes related to the research question, and clusters of codes were identified to represent patterns in the data on a latent level (by interpreting emerging patterns and examining the underlying ideas and assumptions). Saturation was not used as a criterion to limit recruitment; however, we believe that saturation was reached for the themes in this study.

The frequency labels of general, typical, and variant were used to indicate the representativeness of our findings and the recurrence of themes (Hill et al., 2005). The main themes were all general, which means that they applied to all or all but one case, and are referred to as *all participants* in the text. The themes were considered typical if they applied to more than half the cases, which has been referred to as *most participants* in the text. Variance in the themes was defined as being represented by less than half but more than four cases, labeled as *some participants*, while the themes that applied to one to three participants have been referred to as *a few* in the text.

Reflexivity was emphasized throughout the data analysis (Dodgson, 2019). Prior to and during the research process, we reflected on our potential preconceptions and how they could influence the research and the way of understanding and interpreting the parents’ distinctive experiences*.* It is equally important to note that our knowledge and experience of ichthyosis helped us identify areas during the interview that merited further probing. To enhance the trustworthiness of this study, four steps were undertaken (Elo et al., 2014): Credibility checks were performed during each interview. At the end of the interview, the participant was asked whether there was anything that was important to them that had not come up as a topic or a question during the interview. Member checking was carried out by discussing the findings with two members (both parents) of the study’s reference group, and they were encouraged to assess whether the results captured the essence of the parents’ everyday life with regard to caring for a child with ichthyosis. Further, investigator triangulation was used to confirm findings and different perspectives (Carter, Bryant-Lukosius, DiCenso, Blythe, & Neville, 2014). Excerpts from the interviews have been presented to provide transparency in the analytical process and ensure the credibility of the results. F represents ‘father,’ and M indicates ‘mother.’

## Results

Our analysis yielded four main themes: Parents’ and others’ reactions to the child’s difference, Experiences with healthcare services, It’s all about skin care, and Impact on relationships. Table 2 shows the main themes and subthemes. Information about potential differences between parent’s experiences when the child has a mild compared to more severe ichthyosis will be provided in the Results.

**Table 2.** Main themes and subthemes.

Main themes	Subthemes
Parents’ and others’ reactions to the child’s difference	There is something wrong with my baby Perceived stigma
Experiences with healthcare services	Lack of knowledge among healthcare professionals and allied caretakers Lack of guidance and holistic follow-up Hampered communication with healthcare professionals
It’s all about skin care	Touching the skin Lubrication and exfoliation Itching and pain Extensive planning Financial and time burdens
Impact on relationships	Closeness and emotional attachment Lack of privacy Siblings and family planning Hampered communication with partners

## Parents’ and others’ reactions to the child’s difference

This theme describes the parents’ experiences when they notice there is something wrong with their child’s skin, and how they perceive being stigmatized as a result of the child’s different appearance.

### There is something wrong with my baby

All the parents of children with severe forms of ichthyosis noticed that the child’s skin looked different shortly after birth. Some of the infants were covered in unusually thick vernix caseosa (a waxy white substance coating the skin of newborns) that did not wash off easily, while others were covered in a cellophane-like membrane, which made the skin look too tight. A few did not have any skin on large segments of the body. Some parents used words such as ‘skinless,’ ‘alien-like,’ ‘sausage skin,’ ‘fiery red,’ ‘gladpack-ish,’ and ‘white as a sheet’ to describe their children’s skin as newborns. They reported feeling afraid, alone, helpless, and in denial.

Straight after birth, I got my son on my chest; but they removed him shortly after because they saw something was wrong … and so did we; we kind of knew what a newborn should look like … so, he came out and was fiery red. He wasńt blueish white; he was fiery red … and he had, it looked kind of like plastic wrap, on the finger joints. (M)

Parents who observed that the child was in pain or had their baby admitted to the neonatal intensive care unit (NICU) reported feeling high levels of distress. Some parents were afraid that their baby was stillborn or that he/she was going to die.

When she was born, I was 100% sure that she was stillborn because she looked incredibly strange. She actually looked like an alien. (F)

The parents described experiencing depressive symptoms and anxiety when they recalled the initial months after having their baby. A few parents, of children with mild and severe ichthyosis, including a father, reported having a postpartum depression, for which they had to consult a psychologist/therapist.

### Perceived stigma

This subtheme refers to how parents sensed or felt that other people held negative attitudes or beliefs toward the child or its’ condition. A few participants reported that it was emotionally difficult for them to take the baby out in public because of the child’s different appearance. They found it hard when people looked at the infant and this made the parents cover the baby with a blanket or they felt obliged to explain why the child looked different. Parents, of both mildly and severely affected children, experienced staring and questions from others. A few parents of children with severe ichthyosis had experienced being reported to the Child Protection Services by local healthcare providers or their child’s school. The reports described a child who was not cared for properly, smelled bad, and looked dirty, or a child who was difficult to calm due to pain. The issue of attachment disorder was also raised. These reports made the parents doubt themselves as caregivers and affected them negatively. Some of the parents reported that the smell from the skin or the creams became a problem, as the child grew older. Other parents reported how adolescents with ichthyosis used too much perfume to dull the smell because their clothes smelled of rancid lubrication. A few parents described that they pictured the child’s future negatively and had distressing thoughts about the child’s future experiences of kindergarten, school, friendship, romantic relationships, and job opportunities. They assumed that these life events would probably be negatively affected by the ichthyosis. Most parents experienced that the child was stigmatized. A few children had experienced being named ‘old man’, ‘monster’, ‘snake skin’, and ‘disgusting’. Some of the parents reported that their children had experienced other children to be hesitant to hold their hands due to fear of contagion.

## Experiences with healthcare services

The parents reported a general lack of knowledge about ichthyosis in the healthcare system, which could result in a delayed diagnosis, lack of information and follow-up, and misunderstandings.

### Lack of knowledge among healthcare professionals and allied caretakers

When the parents noticed that there was something wrong with their baby’s skin immediately after birth and before the child was diagnosed, they turned to healthcare professionals for answers about what was wrong. Most parents stated that they did not receive any answers because the healthcare professionals did not know. This was a highly stressful experience for the parents. They used words such as ‘frightening,’ ‘shocking,’ ‘surreal,’ and ‘terrifying’ to describe their emotional reactions to the lack of information.

And the frightening thing back then was that the midwives, or the midwife and her assistants, didn’t … did not recognize it … They see thousands of children being born, so when they said, ‘We do not know what this is’ – that was pretty frightening. (F)

A few of the children with severe ichthyosis were transferred to a hospital with national responsibility and expertise on skin disorders, for the child to be diagnosed. One couple experienced that not even specialized healthcare professionals could diagnose their baby with certainty, they felt extremely alone, worried, and scared.

There I was, with the so-called expertise in Norway, and not even they had seen it before, making me feel small and scared. (M)

The timing of a diagnosis varied. Some children were diagnosed before leaving the hospital, others were diagnosed later. Certain parents of children with a milder form of ichthyosis reported that their child’s symptoms appeared gradually, which led to a delay in receiving the correct diagnosis.

### Lack of guidance and holistic follow-up

Following the child’s birth, some of the parents of children with severe ichthyosis, reported receiving counseling from healthcare professionals regarding how to take care of their baby’s skin and the wounds. However, parents did not remember receiving any guidance regarding touching, how to give alternative care, cuddle, and affection to their newborn when skin-to-skin contact was challenged. They all described how they wanted – and needed – more guidance on how to cope with their feelings and worrying thoughts. Some of the parents expressed that they were anxious about touching their baby’s skin due to its different appearance and the fear of inflicting pain on the infant, making them feel insecure and emotionally distressed. The focus of the healthcare professionals was the child and the practical component of ichthyosis, and healthcare professionals did not give potential parental needs any attention.

### Hampered communication with healthcare professionals

Some of the parents of the children with severe ichthyosis, described being emotionally overwhelmed by their child’s diagnosis and felt unable to communicate their worries and everyday situation entailed by the condition to healthcare professionals. A few parents noted that their communication with healthcare professionals could have been hampered by misunderstandings experienced by either party and by healthcare professionals’ reliance on medical jargon. A few parents stated that the doctors asked how the child was doing, but showed no interest in the family or the couple. Thus, the parents were not able to convey how tired they actually were.

Both my husband and I had a breakdown, which I think could have been avoided if we had kind of talked about how we were coping and not only about the child. (M)

During the interview, all parents were asked whether their child had ever been affected by pain, and what kind of counseling they had received in this matter from healthcare professionals. None of the parents recalled healthcare professionals guiding them regarding how to identify whether their baby was in pain.

## It’s all about skin care

This theme highlights how the parents experienced skin care. Parents of severely affected children appeared to suffer from additional distress, due to more extensive and time consuming care needs which could be painful to the child.

### Touching the skin

Most parents, irrespective of disease severity, reported that their child’s skin felt different to the touch compared to normal skin. Some described that touching the skin resembled touching tracing paper or cigarette paper, or they called the skin ‘bumpy,’ ‘fish skin,’ or ‘different.’ Most parents did not feel or share any disgust when touching/looking at their child’s skin, while a few parents did, which could trigger emotional reactions.

I just think, I thought it was gross … when it was … when he had … just returned home, and this layer, outer layer loosened, and the mother peeled it off; it was like … I couldn’t eat … I do not want to use the word disgust, but almost … when it comes to getting used to the fact that there is a lot of skin to peel off. (F)

Some of the parents of children with severe ichthyosis reported having reduced skin contact due to the child feeling pain and being covered in large amounts of cream. Some of the parents kept a layer of fabric between their and their child’s skin to prevent the cream from transferring to their clothes or surroundings. A few parents described hugging and kissing their child less during the newborn stage due to the baby being covered in lotion. The child’s face would be completely covered with cream; the parents wanted to hug the baby, but did not want fat from the cream on their own faces. One father stated that he planned his hugs carefully and kissed the child’s hair instead.

You have to kind of plan your cuddling (…). A thought runs through my head that I kind of have to … if I hug him there, I won’t have to wash my hands or my face afterwards. (F)

### Lubrication and exfoliation

Once the families were discharged from hospital, skin care was the parents’ responsibility. All the parents of the children with the most severe types of ichthyosis had to apply cream/lotion to their child several times a day. Some parents described doing this every two or three hours, and they had to change their child’s diaper and clothes every time. Some of the children enjoyed skin care, while it was painful for others.

All parents reported that the skin care was time consuming, and parents of children with severe forms of ichthyosis stated that they spent hours in the bathroom daily. Some parents adopted strict routines to cope with their child’s skin care, sharing the responsibility between them. Almost all of the affected children needed a bath every day. After soaking in the tub for a while, the majority needed their wet skin to be exfoliated in order to remove the scales that would develop. Most of the parents who had to exfoliate their child’s skin as a part of skin care found it emotionally disturbing because the child cried, protested, or experienced it as painful. A few parents said that they always felt like they should have exfoliated their child’s skin more, but did not because they were afraid that the child would get hurt or that it would deteriorate their relationship with them. One mother stated that others could hardly be inside the house during skin care sessions, because the child howled and screamed so terribly. In order to cope with this, she had to repress her emotions:

I blocked out all emotions, everything, absolutely everything … but I think I did not manage to realize it there and then, to appreciate (my state of mind) in the midst of all these painful procedures … (M)

### Itching and pain

All parents reported itching as a problem and described the children as being desperate to scratch their skin. Some parents found it difficult to sleep at night because they would have to keep listening, to check whether the child was scratching themselves. Some children scratched themselves until they started bleeding, making skin care even more demanding.

Most parents discussed their child’s pain, and they experienced it as an emotional burden. Most participants of children with severe ichthyosis worried about inflicting pain on their newborn baby when touching it and taking care of its skin. A few of the babies had lost much of the skin during birth, leading to big wounds and immense pain.

He experienced lots and lots of pain. There I was, supposed to change his diaper, and he had no skin down below, so he went into an upward bow, foaming at the mouth, and there I was, all alone, not knowing how to handle it. (M)

Some parents of children with severe ichthyosis described how they struggled to find alternative ways to care for, show affection to, or cuddle with their child in order to avoid causing them pain. Healthcare professionals had told one mother to avoid picking up her baby unless necessary because close contact caused pain. This made her feel helpless and unable to take care of her baby’s needs.

I remember thinking, does he need me at all? I didn’t need to be with him, since I felt there was nothing I could do. What was I supposed to do? Just sit there, watching him? It was really hard. (M)

### Financial and time burdens

Some of the parents reported experiencing financial burdens. They reported increased expenses on clothing due to having to replace clothes that were soiled by the creams and lotions used for their child’s skin care. The skin care, housework, and planning were so time consuming that some parents stated that their everyday life was incompatible with having a full-time job.

I’ve never worked full-time hours after I had her. I’ve been working part-time hours due to the heavy workload at home with her … It’s been more than enough to deal with. (M)

Most parents reported that there were several aspects of ichthyosis that were time consuming. Skin care was one of these, taking several hours daily. Another time consuming component was housework. Parents reported that their child left a lot of dandruff in the house, which meant that vacuuming and the changing of bed linen were needed frequently. A few parents described that their child had a specific smell that tainted their bed and clothes, which led the parents to change mattresses regularly.

Where she has been sitting, it looks like it’s been snowing … There’s a huge amount of skin scales. (F)

Multiple daily changes of clothes combined with clothes becoming stiff and saturated with cream/ointment led to a high amounts of laundry. Some parents of children with severe ichthyosis had bought a separate washing machine – in some cases, an industrial model – so that the affected child’s cream-filled clothes could be washed separately. A few parents reported that the machine’s pipes and drains needed cleaning once or twice a month to prevent them from clogging due to the cream and skin flakes.

### Extensive planning

All parents reported having to plan extensively due to the ichthyosis and being unable to go anywhere without the creams and extra clothes. A few specifically mentioned the challenges involved with carrying out the skin care sessions anywhere outside their home when the child was younger. They always had to bring towels, and two adults would be required while lubricating the child’s skin to prevent the cream from spreading to the surroundings.

The amount of planning did not diminish over time, as the child grew older. During overnight school trips, a few parents of children with severe ichthyosis described how they would have to pick up the child, take them home to be bathed and to lubricate their skin, and then drive them back. Other parents reported that they did not go on vacations to warmer regions due to their child’s problems with regulating heat. If they went on holidays, they had to figure out how much cream the child would need for the entire stay, in order to pack and bring the necessary amount.

More of everything. It’s more care, more changes of clothes, more laundry. That is, whether we are going to the cabin or abroad, there is … a giant suitcase of clothes for him. (F)

## Impact on relationships

The fourth theme describes how the massive daily focus and work associated with ichthyosis, affected the relationships within the families.

## Closeness and emotional attachment

Many of the parents, irrespective of disease severity, expressed that the required skin care provided a unique opportunity for establishing a close relationship with their child and reported feeling deeply attached to it, in spite of their initial emotional reactions to the child’s skin condition. Most mothers succeeded in breastfeeding. Many of the parents had actively decided early on that they would try to transform skin care into a pleasant experience for both parties.

I have asked myself whether having a child with a different kind of skin … if it can make me feel some kind of disgust … (…) I may have felt it in the beginning without realizing it … (…) because when you see such cracked fish skin, it’s not pretty … At the same time, I think that the fact that I had to lubricate her skin so much and that we had to talk so extensively about the skin might have strengthened my attachment to her. It is possible that you get this special contact precisely due to the lubrication. (M)

Irrespective of disease severity, other parents did not feel that the diagnosis had affected their attachment or relationship with the child, neither positively nor negatively.

### Lack of privacy

Parents of children with both mild and severe ichthyosis, felt that the child’s boundaries were challenged as the child grew older because of the intimate nature of skin care. Some children were not interested in lubricating their skin as often as the parents thought necessary, and their skin therefore looked drier and more different than when the parents were in charge. The parents struggled with accepting their child’s choice of reduced skin care, worrying that a different appearance would lead to stigmatization, gazes, and questions. A few parents of children with both mild and severe ichthyosis, stated that skin care became difficult and more time consuming as their child grew older because the child did not cooperate when lubricating the skin. The parents also felt that their relationship with the child was challenged, as a consequence. Skin care, thus, became an extra struggle, making it difficult for the parent to reach work on time; a few even had to reduce their working hours or quit their job.

### Siblings and family planning

Most of the children with ichthyosis had unaffected siblings. Parents stated that they spent a significant amount of time taking care of the affected siblings’ skin, leaving less time for the unaffected siblings. A few reported that they did not worry about their relationship with the child with ichthyosis; instead, they worried about the child’s siblings who received far less attention and time with them. Parents described how ichthyosis could be all-consuming for a family, creating a risk of unaffected children becoming ‘invisible’ and weakening their relationship with these children.

I have started to wonder if it has been too much of ichthyosis. I must have done something wrong. It should not be like this. I have lost a child (sibling without ichthyosis). I truly have. (M)

Some parents disclosed troubling thoughts during subsequent pregnancies after having a child with ichthyosis.

So much revolved around our first child with ichthyosis. It would be so unfair for our child to have a sibling without ichthyosis, but we don’t want another child with the same challenges … And yes, I remember it was really difficult. There were a lot of thoughts. (M)

### Hampered communication with partners

Some of the parents found it difficult to talk openly about what they felt with their partner. A few fathers reported thinking that the mothers had enough of their own grief, and, thus, chose not to bother them with their own worrying thoughts. As a result, they were alone with their worries. Mothers did not report this.

The first time (after birth), I did not feel like holding him. I was afraid he would get hurt … I don’t think I told my wife about it … I think she was having an even harder time than me … I felt she had more than enough to think about. (F)

## Discussion

The aim of the present study was to explore parents’ experiences with raising a child with ichthyosis, using a qualitative methodology. The parents described parenthood at odds with their prior expectations, and additional caregiver burdens associated with their child’s diagnosis. They reported how the affected child had an extensive need for help and assistance from them during childhood, first of all due to daily skin care needs. This could affect relationships within the family. Very few studies have investigated how parents experience raising a child with ichthyosis, and the challenges they experience in everyday life. Therefore, these novel findings highlight a considerable number of challenges and issues that were of central importance to the parents, and could guide healthcare professionals with respect to where they should focus their efforts when following-up a family affected by ichthyosis.

### When complex conditions affect daily skin contact

The parents of children with severe forms of ichthyosis expressed feeling concerned and anxious when touching their baby’s skin for the first time after birth. This is a disquieting finding and in sharp contrast to most mothers’ experiences. Normally, after a child is born, mothers will immediately start to engage in typical maternal behaviors, such as cradling their infant, paying attention to its face and body, and providing it with an affectionate touch (Feldman, 2011). Touch is the most basic mammalian maternal behavior and plays an important role in children’s socio-emotional development (Bystrova et al., 2009; Feldman, 2011). Kangaroo care, the method that involves prolonged skin-to-skin contact, was developed in Columbia in the 1970s for reducing mortality among preterm babies (Charpak et al., 2017). It is said to improve sleep, neurodevelopment, and growth in infants, which demonstrates the importance of early skin-to-skin contact (Campbell-Yeo, Disher, Benoit, & Johnston, 2015).

Our study indicates that some of the parents of children with severe forms of ichthyosis had less or impaired skin-to-skin contact with their babies initially after birth, due to the child’s appearance and/or the fear of potentially causing pain. This is in line with parents of children with osteogenesis imperfecta, who also report fear of injuring their child or causing pain, and therefore, as a consequence, limit cuddling and physical contact (Deatrick, Knafl, & Walsh, 1988). Similarly, parents of children with epidermolysis bullosa, also share the challenging emotional impact of witnessing their child in pain (Kearney et al., 2020). In our study, parents described some factors that could possibly affect parental touch-related behaviors, like for instance experiencing depressive symptoms, feeling anxious and shocked, and displaying high levels of psychological distress, as they understood that there was something wrong with their newborn child. Postpartum depression may also reduce one’s capacity for physical proximity or affectionate touch (Feldman, 2011), and was reported by a few parents. Children with the severe forms of ichthyosis can have reduced facial mimics due to stretched skin, which could make it even more of a challenge for the parents to see or interpret their child’s emotions and might potentially hinder parent–child interaction further.

The parents in our study had to perform skin care on their child, including washing, exfoliating, and lubricating the skin, which involves skin-to-skin contact several times a day. However, understandably, demanding skin care was not perceived as an ordinary part of caring for their baby or child, but as an extra caregiver burden. Some parents of children with mild ichthyosis, or parents whose children did not suffer from pain during skin care, did state that they had managed to transform skin care sessions into quality time and a positive experience, because of the close contact it created with their child.

### Ichthyosis and parent–child interaction

A child’s development is shaped by every day’s countless small exchanges and interactions with parents (Bruschweiler-Stern, 2009). Parents need to understand the baby’s way of communicating, regulate and answer the child’s needs, and establish a relationship with it. When parents feel able in fulfilling these tasks, coping with parental demands is experienced (Bruschweiler-Stern, 2009). Interaction and social exchanges may be disrupted when the child is born with ichthyosis. It may be difficult for the parents to identify and fulfill the child’s needs due to pain, which makes the child hard to soothe. This may lead to parents doubting their parental skills, and trigger anxiety (Bruschweiler-Stern, 2009). Children need positive interactions, but establishing positive interaction patterns may be challenging when a child is born with ichthyosis. The theoretical and empirical literature has described the possible influence of chronic pain in the establishment of parental relationships (Donnelly & Jaaniste, 2016). Disease, and particularly pain, may trigger an increased need for secure relationships and a close, caring other (Donnelly & Jaaniste, 2016). Hence, children tend to seek support in their parents when exposed to a threatening situation, such as for instance when experiencing pain. However, when the child is affected by ichthyosis, parents are the ones exposing them to pain, hence depriving the child of the comforting parent in painful situations. This could create distress in parents, and demanding emotional and behavioral dynamics between children and parents, as has been described also by parents of children with epidermolysis bullosa and other complex chronic conditions (Kearney et al., 2020; Miller et al., 2009), which may require provision of support or psychological interventions. However, most of the parents in our study reported to cope well, although some of them felt uncertainty regarding touching their child and finding alternative ways of showing affection and love for it. They also reported how moments of meeting and early interactions were disrupted by their feelings of apprehension due to the visibly different skin and obvious pain in some of the children.

### Parental stress factors and resilience

All the parents of children with severe form of ichthyosis noticed that there was something wrong with their child’s skin immediately after birth. They expressed shock and fear associated with adapting to new roles, having to reorganize their lives and expectations, and coping with the increased care demands. This has also been described by parents who have a child with other visible congenital conditions, such as cleft lip and palate or epidermolysis bullosa (Nelson, Glenny, Kirk, & Caress, 2012; Wu, Sun, & Lee, 2020). Parents of children who have a rare disorder may face the challenges of a delayed diagnosis, a lack of peer support, and insufficient knowledge among healthcare providers (Jaffe, Zurynski, Beville, & Elliott, 2010). Parents may experience unmet health and social needs, which may lead to increased physical and emotional distress (Currie & Szabo, 2019). In our study, parents’ feelings of worry, frustration, and distress were found to be intensified by a delayed diagnosis for parents of children with mild ichthyosis, and the lack of relevant knowledge among healthcare professionals, in line with prior research (Currie & Szabo, 2019). Parents also experienced other stress factors, such as extensive skin care, pain in their child, a different appearance of the skin, and perceived courtesy stigma. Most families in our study adapted well, in spite of the many challenges in their daily lives. Parental resilience is defined as ‘the capacity of parents to deliver competent, quality parenting to children despite adverse personal, family, and social circumstances’ (Gavidia-Payne, Denny, Davis, Francis, & Jackson, 2015, p. 113). In our study, the birth of a child with ichthyosis clearly brought adversity and hardship in many parents’ lives, indicating that parental resilience may be a relevant construct within research on complex skin conditions and should be followed-up by future research. A number of factors, like for instance parents’ psychological functioning, self-efficacy, family functioning, and social connectedness may play a protective role for the development and maintenance of parental resilience (Gavidia-Payne et al., 2015). Future studies should address why some parents of children with skin disorders like ichthyosis thrive and respond positively to the situation, while others do not.

### Skin care, self-care, and autonomy

Some of the parents in our study described continuing with skin care also with their older children, as they struggled in the shifting process of management responsibility from parent to child. In some cases, they did not share the child /adolescent’s view on condition management. The child did not want to lubricate their skin as often as their parents did when they were in charge, which had an impact on the skin’s appearance. As a consequence, parents worried about stigmatizing and people staring.

One core dimension of positive parenting is the encouragement of the child’s autonomy (Steinberg, 2010). According to our findings, autonomy was challenged in some families, because of the child’s need for some degree of assistance with skin care throughout their lives. The transition to autonomy for children/adolescents with a chronic illness poses a challenge for other diagnoses than ichthyosis, as demonstrated in a recent systematic review (Lerch & Thrane, 2019). In case of congenital ichthyosis, skin care is a parent-driven effort when the child is young. The goal is that the learning of self-care practices should become child-driven as the child grows older. To ease this process and include the child as an active participant, they should be involved in decision-making processes depending on their developmental stage. At the same time, parents also need to hand over some responsibility and control to the child and support the child in its attempts to autonomously shoulder the responsibility of their own skin care. A prerequisite for developing self-care is support, assistance, and advice from parents and healthcare professionals. Hence, healthcare professionals should help parents communicate disease-specific knowledge and symptom assessment processes to their child/adolescent (Lerch & Thrane, 2019). Further, the early involvement of professional caregivers to help transfer knowledge regarding skin care should be prioritized, to reduce an unnaturally prolonged parental responsibility. Few studies have studied the shifting of management responsibility, and most of the studies have been conducted on parents and adolescents with diabetes (Kayle, Tanabe, Shah, Baker-Ward, & Docherty, 2016). Future studies should address this gap in knowledge in complex skin diseases.

### Stigma and disgust

Stigma describes negative attitudes or discrimination against someone based on a discredited attribute (Goffman, 1963). Stigma theory also describes how experiences of stigma may spread from the stigmatized individual to its close connections, known as courtesy stigma (Goffman, 1963), and reported by parents in the present study. Skin is a highly visible organ, and may communicate important information about a persons’ health (Wu & Cohen, 2019). A few parents experienced stigma when they were judged as neglectful caregivers due to smell or the appearance of the child’s skin. This made the parents feel helpless and insecure about their own ability to raise the child with ichthyosis. Parents described how they tried to cover the child’s skin, helped their children with skin care in order to reduce difference and keep the skin’s appearance as normal as possible. Parents hoped that this would reduce staring and comments from other people, and avoid the children experiencing stigmatization as well.

A few parents also stated that they felt disgust when first getting to know their child. Broken skin (skin that has fissures, cracks, bleeds etc.), such as in ichthyosis, may elicit disgust (Clarke, Thompson, & Norman, 2020), a concept that is understudied in research in the dermatologic field (Mento, Rizzo, Muscatello, Zoccali, & Bruno, 2020). Within clinical care, healthcare professionals may not feel confident in exploring this topic with parents or individuals with skin disorders such as ichthyosis. Potential feelings of disgust may also be difficult to share with others. Hence, parents may struggle with difficult feelings without support from health care services or others. As demonstrated also in parents of children with epidermolysis bullosa, the social and emotional impact of complex skin conditions on parents is understudied, and need to be addressed by appropriate support within health care services (Kearney et al., 2020; Wu & Cohen, 2019).

### Pain

When asked directly, most parents of severely affected children reported pain being a challenge, especially during skin care sessions, and in some cases developing into a daily struggle between the child and the parent as the child grew older. In some cases, the struggle resulted in parents physically holding their child for exfoliation and lubrication, which could potentially hamper their relationship with the child and negatively impact their own psychological health. None of the parents remembered being informed by healthcare professionals that infants with ichthyosis could experience skin-related pain. Pain in newborns can have negative short- and long-term outcomes and must therefore be addressed and alleviated (Eriksson & Campbell-Yeo, 2019). When parents’ pain assessment is the basis of pain management, it is important to examine whether they are able to perform pain assessment properly (Olsson, Pettersson, Eriksson, & Ohlin, 2019; Xavier Balda et al., 2000). Studies on older children show that parents may tend to underestimate a child’s acute or postoperative pain, which, in turn, leads to a risk of under-treatment (Brudvik, Moutte, Baste, & Morken, 2017). Many parents in our study reported that their children experienced pain during skin care, but none of them mentioned anything about analgesic treatment, which could indicate that this was an unaddressed issue. Parents of children with ichthyosis should receive guidance on how to detect and reduce the pain experienced by their child, and how to cope with this emotionally. Support should also be provided when indicated.

## Implications for practice

Healthcare professionals who work with children affected by ichthyosis should assist the parents in trial and error when exploring how to find alternative ways of handle, care for, and show affection for their infant from birth when touching may be associated with pain, or hampered due to greasy ointment. The parents may also need help to understand the baby’s efforts of communicating. Healthcare professionals should be aware of, and inform parents, that infants with ichthyosis may feel pain, and help parents to recognize the baby’s signals. The parents should also be guided on available and suitable analgesic treatment.

In the classification of psychodermatological disorders, ichthyosis is classified as a condition that is associated with symptoms of psychological distress (Patel & Jafferany, 2020). This is demonstrated in our study as well, from the parents’ point of view. Hence, it is important to identify parents at risk and in need of support, so that help can be offered and tailored to their specific needs.

As the child with ichthyosis grows older, healthcare professionals should include the children in decision making processes regarding skin care, to gradually enhance and empower their readiness for self-care, and preparing the parents for their child’s growing autonomy. Healthcare professionals are advised to assess the parent–child interaction, and their progress in shifting the management responsibility of skin care. Consultations need to facilitate discussions between parents and child, to arrive on a consensual understanding of which challenges they meet in shifting the responsibility, and address coping strategies to deal with them.

Parents of the current study experienced courtesy stigma, and also reported that they thought their children felt stigmatized. It is important that healthcare professionals address issues of stigma with parents of children with ichthyosis, to assess how parents cope with other people’s questions and staring. Parents who experience difficulties with stigmatization, should be offered tailored psychological interventions and counseling.

The management of ichthyosis also needs to be integrated within the broader family context, as the degree of care needed for children with severe forms of ichthyosis may impact all family members. Healthcare professionals should strive to offer interventions that promote siblings’ emotional and psychological needs (Haukeland et al., 2020).

## Strengths and limitations

The current study provides a unique insight into parents’ experiences of caring for a child with ichthyosis. Some limitations, however, should be considered when interpreting the results. The interviews were conducted over the phone, which hampered the interviewer’s ability to attend to nonverbal cues (Holt, 2010). Video calls or face-to-face interviews would have enabled visual cues. On the other hand, the participants may have preferred the sense of anonymity provided by telephone calls, particularly when discussing sensitive topics (Heath, Williamson, Williams, & Harcourt, 2018). In addition, telephone interviews facilitated the participation of those who would otherwise have been hard to reach (Drabble, Trocki, Salcedo, Walker, & Korcha, 2016).

The majority of participants were recruited through the voluntary registry of the CRD, and the sample may therefore not be totally representative of the population that was studied. Another limitation was that participation rate was 48% for parents of children under 16 years of age, and only 11% of those over 16 years of age, which could potentially bias findings**.** All the children of the participating parents had ichthyosis, but there was heterogeneity in terms of disease severity, which introduces variations in treatment and the impact that the condition has on their daily life. Due to the rarity of ichthyosis, the limited number of potential participants also led to the inclusion of patients of a wide age range. Some of them are now adults, and the practices in health care may have changed since they were born. The wide age range could also possibly have led to a recall bias, but interestingly we found that parents of older children still vividly recalled strong and challenging feelings and experiences, in line with what was shared by the parents of the younger children. In order to check for relevance of findings, results were checked with the project’s reference group, who confirmed that the findings represent important and well-known challenges that are familiar to parents of children with ichthyosis and also stated that results may offer valuable insight into the experiences related to caring for these children.

## Conclusion

The current study explored parents’ experiences of caring for a child with ichthyosis. The parents of children with complex skin conditions, such as ichthyosis, face a number of challenges with regard to caring for their child, in addition to the shock of having a baby with a rare skin condition. Healthcare professionals responsible for the follow-up of families affected by ichthyosis need to be aware of the extent of skin care required, its consequences on parental caregiving experiences, and how a diagnosis of ichthyosis affects the whole family. We recommend a multidisciplinary and holistic approach to healthcare to attend to this group of parents’ needs.

## References

[CIT0001] WHO immediate KMC Group, Arya, S., Naburi, H., Kawaza, K., Newton, S., Anyabolu, C. H., … Massawe, A. (2021). Immediate “kangaroo mother care” and survival of infants with low birth weight. *The New England Journal of Medicine*, *384*(21), 2028–2038. doi:10.1056/NEJMoa2026486 34038632 PMC8108485

[CIT0002] Bodemer, C., Bourrat, E., Mazereeuw-Hautier, J., Boralevi, F., Barbarot, S., Bessis, D., … Sibaud, V. (2011). Short- and medium-term efficacy of specific hydrotherapy in inherited ichthyosis. *British Journal of Dermatology*, *165*(5), 1087–1094. doi:10.1111/j.1365-2133.2011.10510.x 21729027

[CIT0003] Braun, V., & Clarke, V. (2006). Using thematic analysis in psychology. *Qualitative Research in Psychology*, *3*(2), 77–101. doi:10.1191/1478088706qp063oa

[CIT0004] Braun, V., & Clarke, V. (2019). Reflecting on reflexive thematic analysis, Qualitative research. *Sport, Exercise and Health*, *11*(4), 589–597. DOI:10.1080/2159676X.2019.1628806

[CIT0005] Brudvik, C., Moutte, S. D., Baste, V., & Morken, T. (2017). A comparison of pain assessment by physicians, parents and children in an outpatient setting. *Emergency Medical Journal*, *34*(3), 138–144. doi:10.1136/emermed-2016-205825 PMC5502236 27797872

[CIT0006] Bruschweiler-Stern, N. (2009). The neonatal moment of meeting—building the dialogue, strengthening the bond. *Child and Adolescent Psychiatric Clinics of North America*, *18*(3), 533–544. 19486836 10.1016/j.chc.2009.02.001

[CIT0007] Bystrova, K., Ivanova, V., Edhborg, M., Matthiesen, A. S., Ransjo-Arvidson, A. B., Mukhamedrakhimov, R., … Widstrom, A. M. (2009). Early contact versus separation: Effects on mother-infant interaction one year later. *Birth (berkeley, Calif)*, *36*(2), 97–109. doi:10.1111/j.1523-536X.2009.00307.x 19489802

[CIT0008] Campbell-Yeo, M. L., Disher, T. C., Benoit, B. L., & Johnston, C. C. (2015). Understanding kangaroo care and its benefits to preterm infants. *Pediatric Health, Medicine and Therapeutics*, *6*, 15–32. doi:10.2147/PHMT.S51869 PMC5683265 29388613

[CIT0009] Carter, N., Bryant-Lukosius, D., DiCenso, A., Blythe, J., & Neville, A. J. (2014). The use of triangulation in qualitative research. *Oncology Nursing Forum*, *41*(5), 545–547. doi:10.1188/14.Onf.545-547 25158659

[CIT0010] Charpak, N., Tessier, R., Ruiz, J. G., Hernandez, J. T., Uriza, F., Villegas, J., … Maldonado, D. (2017). Twenty-year follow-up of kangaroo mother care versus traditional care. *Pediatrics*, *139*(1), 1–11. doi:10.1542/peds.2016-2063 27965377

[CIT0011] Chuong, C. M., Nickoloff, B. J., Elias, P. M., Goldsmith, L. A., Macher, E., Maderson, P. A., … Christophers, E. (2002). What is the ‘true’ function of skin? *Experimental Dermatology*, *11*(2), 159–187. doi:10.1034/j.1600-0625.2002.00112.x 11994143 PMC7010069

[CIT0012] Clarke, E. N., Thompson, A. R., & Norman, P. (2020). Depression in people with skin conditions: The effects of disgust and self-compassion. *British Journal of Health Psychology*, *25*, 540–557. doi:10.1111/bjhp.12421 32394487

[CIT0013] Cousino, M. K., & Hazen, R. A. (2013). Parenting stress among caregivers of children with chronic illness: A systematic review. *Journal of Pediatric Psychology*, *38*(8), 809–828. doi:10.1093/jpepsy/jst049 23843630

[CIT0014] Currie, G., & Szabo, J. (2019). “It is like a jungle gym, and everything is under construction”: The parent’s perspective of caring for a child with a rare disease. *Child: Care, Health and Development*, *45*(1), 96–103. doi:10.1111/cch.12628 30370696

[CIT0015] Deatrick, J. A., Knafl, K. A., & Walsh, M. (1988). The process of parenting a child with a disability: Normalization through accommodations. *Journal of Advanced Nursing*, *13*(1), 15–21. doi:10.1111/j.1365-2648.1988.tb01387.x 3372881

[CIT0016] DiCicco-Bloom, B., & Crabtree, B. F. (2006). The qualitative research interview. *Medical Education*, *40*(4), 314–321. doi:10.1111/j.1365-2929.2006.02418.x 16573666

[CIT0017] Dodgson, J. E. (2019). Reflexivity in qualitative research. *Journal of Human Lactation*, *35*(2), 220–222. doi:10.1177/0890334419830990 30849272

[CIT0018] Donnelly, T. J., & Jaaniste, T. (2016). Attachment and chronic pain in children and adolescents. *Children*, *3*(4), 21. 10.3390/children3040021 PMC5184796 27792141

[CIT0019] Drabble, L., Trocki, K. F., Salcedo, B., Walker, P. C., & Korcha, R. A. (2016). Conducting qualitative interviews by telephone: Lessons learned from a study of alcohol use among sexual minority and heterosexual women. *Qualitative Social Work: Research and Practice*, *15*(1), 118–133. doi:10.1177/1473325015585613 PMC4722874 26811696

[CIT0020] Dreyfus, I., Pauwels, C., Bourrat, E., Bursztejn, A. C., Maruani, A., Chiaverini, C., … Mazereeuw-Hautier, J. (2015). Burden of inherited ichthyosis: A French national survey. *Acta Dermato- Venereologica*, *95*(3), 326–328. doi:10.2340/00015555-1955 25510955

[CIT0021] Dures, E., Morris, M., Gleeson, K., & Rumsey, N. (2011). The psychosocial impact of epidermolysis bullosa. *Qualitative Health Research*, *21*, 771–782. 21343430 10.1177/1049732311400431

[CIT0022] Dyer, J. A., Spraker, M., & Williams, M. (2013). Care of the newborn with ichthyosis. *Dermatologic Therapy*, *26*(1), 1–15. doi:10.1111/j.1529-8019.2012.01555.x 23384016

[CIT0023] Elo, S., Kääriäinen, M., Kanste, O., Pölkki, T., Utriainen, K., & Kyngäs, H. (2014). Qualitative content analysis: A focus on trustworthiness. *SAGE Open*, *4*(1), 1–10. doi:10.1177/2158244014522633

[CIT0024] Eriksson, M., & Campbell-Yeo, M. (2019). Assessment of pain in newborn infants. *Seminars in Fetal and Neonatal Medicine*, *24*(4), 101003. doi:10.1016/j.siny.2019.04.003 30987943

[CIT0025] Feldman, R. (2011). Maternal touch and the developing infant. In M. J. Hertenstein, & S. J. Weiss (Eds.), *The handbook of touch: Neuroscience, behavioral, and health perspectives* (pp. 373–407). New York: Springer Publishing Co.

[CIT0026] Gånemo, A. (2010). Quality of life in Swedish children with congenital ichthyosis. *Dermatology Reports*, *2*(1), e7. doi:10.4081/dr.2010.e7 25386243 PMC4211474

[CIT0027] Gavidia-Payne, S., Denny, B., Davis, K., Francis, A., & Jackson, M. (2015). Parental resilience: A neglected construct in resilience research. *Clinical Psychologist*, *19*(3), 111–121.

[CIT0028] Goffman, E. (1963). *Stigma: Notes on the management of spoiled identity. Simon and schuster*. Englewood Cliffs: Prentice-Hall.

[CIT0029] Gray, D. E. (2018). *Doing research in the real world* (4th ed.). London: Sage.

[CIT0030] Haukeland, Y. B., Czajkowski, N. O., Fjermestad, K. W., Silverman, W. K., Mossige, S., & Vatne, T. M. (2020). Evaluation of “SIBS”, an intervention for siblings and parents of children with chronic disorders. *Journal of Child and Family Studies*, *29*(8), 2201–2217. doi:10.1007/s10826-020-01737-x

[CIT0031] Heath, J., Williamson, H., Williams, L., & Harcourt, D. (2018). “It’s just more personal”: using multiple methods of qualitative data collection to facilitate participation in research focusing on sensitive subjects. *Applied Nursing Research*, *43*, 30–35. doi:10.1016/j.apnr.2018.06.015 30220360

[CIT0032] Hill, C. E., Knox, S., Thompson, B. J., Williams, E. N., Hess, S. A., & Ladany, N. (2005). Consensual qualitative research: An update. *Journal of Counseling Psychology*, *52*(2), 196–205. doi:10.1037/0022-0167.52.2.196

[CIT0033] Holt, A. (2010). Using the telephone for narrative interviewing: A research note. *Qualitative Research*, *10*(1), 113–121. doi:10.1177/1468794109348686

[CIT0034] Jaffe, A., Zurynski, Y., Beville, L., & Elliott, E. (2010). Call for a national plan for rare diseases. *Journal of Paediatrics and Child Health*, *46*(1–2), 2–4. doi:10.1111/j.1440-1754.2009.01608.x 19943870

[CIT0035] Kayle, M., Tanabe, P., Shah, N. R., Baker-Ward, L., & Docherty, S. L. (2016). Challenges in shifting management responsibility from parents to adolescents with sickle cell disease. *Journal of Pediatric Nursing*, *31*(6), 678–690. 27451007 10.1016/j.pedn.2016.06.008

[CIT0036] Kearney, S., Donohoe, A., & McAuliffe, E. (2020). Living with epidermolysis bullosa: Daily challenges and health-care needs. *Health Expectations*, *23*(2), 368–376. doi:10.1111/hex.13006 31868299 PMC7104643

[CIT0037] Kepreotes, E., Keatinge, D., & Stone, T. (2010). The experience of parenting children with chronic health conditions: A new reality. *Journal of Nursing and Healthcare of Chronic Illness*, *2*(1), 51–62. doi:10.1111/j.1752-9824.2010.01047.x

[CIT0038] Lerch, M. F., & Thrane, S. E. (2019). Adolescents with chronic illness and the transition to self-management: A systematic review. *Journal of Adolescence*, *72*, 152–161. doi:10.1016/j.adolescence.2019.02.010 30903932

[CIT0039] Lim, J.-W., & Zebrack, B. (2004). Caring for family members with chronic physical illness: A critical review of caregiver literature. *Health and Quality of Life Outcomes*, *2*, 50–50. doi:10.1186/1477-7525-2-50 15377384 PMC521496

[CIT0040] Mazereeuw-Hautier, J., Hernandez-Martin, A., O’Toole, E. A., Bygum, A., Amaro, C., Aldwin, M., … Oji, V. (2018). Management of congenital ichthyoses: European guidelines of care: Part two. *British Journal of Dermatology*, *13*, 13. 10.1111/bjd.16882 29897631

[CIT0041] Mento, C., Rizzo, A., Muscatello, M., Zoccali, R. A., & Bruno, A. (2020). Negative emotions in skin disorders: A systematic review. *International Journal of Psychological Research*, *13*(1), 71–86. doi:10.21500/20112084.4078 32952965 PMC7498125

[CIT0042] Miller, A. R., Condin, C. J., McKellin, W. H., Shaw, N., Klassen, A. F., & Sheps, S. (2009). Continuity of care for children with complex chronic health conditions: Parents’ perspectives. *BMC Health Services Research*, *9*, 242. doi:10.1186/1472-6963-9-242. PMID: 20025770; PMCID: PMC2805629. 20025770 PMC2805629

[CIT0043] Moola, F. J. (2012). “This is the best fatal illness that you can have”: contrasting and comparing the experiences of parenting youth with cystic fibrosis and congenital heart disease. *Qualitative Health Research*, *22*(2), 212–225. doi:10.1177/1049732311421486 21890712

[CIT0044] Moore, E. R., Bergman, N., Anderson, G. C., & Medley, N. (2016). Early skin-to-skin contact for mothers and their healthy newborn infants. *Cochrane Database of Systematic Reviews*, *11*, 1–121. doi:10.1002/14651858.CD003519.pub4 PMC6464366 27885658

[CIT0045] Nelson, P., Glenny, A. M., Kirk, S., & Caress, A. L. (2012). Parents’ experiences of caring for a child with a cleft lip and/or palate: A review of the literature. *Child: Care, Health and Development*, *38*(1), 6–20. doi:10.1111/j.1365-2214.2011.01244.x 21623872

[CIT0046] O’Brien, B. C., Harris, I. B., Beckman, T. J., Reed, D. A., & Cook, D. A. (2014). Standards for reporting qualitative research: A synthesis of recommendations. *Academic Medicine*, *89*(9), 1245–1251. doi:10.1097/acm.0000000000000388 24979285

[CIT0047] Oji, V., Tadini, G., Akiyama, M., Blanchet Bardon, C., Bodemer, C., Bourrat, E., … Traupe, H. (2010). Revised nomenclature and classification of inherited ichthyoses: Results of the First Ichthyosis Consensus Conference in Sorèze 2009. *Journal of the American Academy of Dermatology*, *63*(4), 607–641. doi:10.1016/j.jaad.2009.11.020 20643494

[CIT0048] Olsson, E., Pettersson, M., Eriksson, M., & Ohlin, A. (2019). Oral sweet solution to prevent pain during neonatal hip examination: A randomised controlled trial. *Acta Paediatrica*, *108*(4), 626–629. doi:10.1111/apa.14588 30246505 PMC6585692

[CIT0049] Patel, A., & Jafferany, M. (2020). Multidisciplinary and holistic models of care for patients with dermatologic disease and psychosocial comorbidity: A systematic review. *JAMA Dermatology*, *156*(6), 686–694. doi:10.1001/jamadermatol.2020.0394 32347896

[CIT0050] Pigg, M. H., Bygum, A., Gånemo, A., Virtanen, M., Brandrup, F., Zimmer, A. D., … Fischer, J. (2016). Spectrum of autosomal recessive congenital ichthyosis in Scandinavia: Clinical characteristics and novel and recurrent mutations in 132 patients. *Acta Dermato- Venereologica*, *96*(7), 932–937. doi:10.2340/00015555-2418 27025581

[CIT0051] Richard, G. (2017). Autosomal recessive congenital ichthyosis. In M. Adam, H Ardinger, & R. A. Pagon (Eds.), *GeneReviews® [Internet]*. Seattle: University of Washington.

[CIT0052] Senger, B. A., Ward, L. D., Barbosa-Leiker, C., & Bindler, R. C. (2016). Stress and coping of parents caring for a child with mitochondrial disease. *Applied Nursing Research*, *29*, 195–201. doi:10.1016/j.apnr.2015.03.010 26856513

[CIT0053] Stabile, M., & Allin, S. (2012). The economic costs of childhood disability. *The Future of Children/Center for the Future of Children, the David and Lucile Packard Foundation*, *22*, 65–96. doi:10.2307/41475647 22550686

[CIT0054] Steinberg, L. (2010). *Adolescence* (9th ed). New York: McGraw-Hill.

[CIT0055] Sun, Q., Ren, I., Zaki, T., Maciejewski, K., & Choate, K. (2020). Ichthyosis affects mental health in adults and children: A cross-sectional study. *Journal of the American Academy of Dermatology*, *83*(3), 951–954. doi:10.1016/j.jaad.2020.01.052 32006604

[CIT0056] Takeichi, T., & Akiyama, M. (2016). Inherited ichthyosis: Non-syndromic forms. *The Journal of Dermatology*, *43*(3), 242–251. doi:10.1111/1346-8138.13243 26945532

[CIT0057] Traupe, H., Fischer, J., & Oji, V. (2014). Nonsyndromic types of ichthyoses: An update. *Journal of the German Society of Dermatology*, *12*(2), 109–121. 24119255 10.1111/ddg.12229

[CIT0058] Troiano, G., & Lazzeri, G. (2020). A review of quality of life of patients suffering from ichthyosis. *Journal of Preventive Medicine and Hygiene*, *61*(3), E374. doi:10.1111/ddg.12229 33150225 PMC7595076

[CIT0059] Vahlquist, A., Fischer, J., & Torma, H. (2018). Inherited nonsyndromic ichthyoses: An update on pathophysiology, diagnosis and treatment. *American Journal of Clinical Dermatology*, *19*(1), 51–66. doi:10.1007/s40257-017-0313-x 28815464 PMC5797567

[CIT0060] Van Scheppingen, C., Lettinga, A., Duipmans, J., Maathuis, C., & Jonkman, M. (2008). The main problems of parents of a child with epidermolysis bullosa. *Qualitative Health Research*, *18*, 545–556. doi:10.1177/1049732308315110 18354052

[CIT0061] Wu, J. H., & Cohen, B. A. (2019). The stigma of skin disease. *Current Opinion in Pediatrics*, *31*(4 ), 509–514. doi:10.1097/MOP.0000000000000792 31188167

[CIT0062] Wu, Y. H., Sun, F. K., & Lee, P. Y. (2020). Family caregivers' lived experiences of caring for epidermolysis bullosa patients: A phenomenological study. *Journal of Clinical Nursing*, *29*(9-10), 1552–1560. doi:10.1111/jocn.15209 32043289

[CIT0063] Xavier Balda, R., Guinsburg, R., de Almeida, M. F., Peres, C., Miyoshi, M. H., & Kopelman, B. I. (2000). The recognition of facial expression of pain in full-term newborns by parents and health professionals. *Archives of Pediatrics and Adolescent Medicine*, *154*(10), 1009–1016. doi:10.1001/archpedi.154.10.1009 11030853

[CIT0064] Yoneda, K. (2016). Inherited ichthyosis: Syndromic forms. *The Journal of Dermatology*, *43*(3), 252–263. doi:10.1111/1346-8138.13284 26945533

